# A special issue on TGF-β signaling and biology

**DOI:** 10.1186/2045-3701-1-39

**Published:** 2011-12-28

**Authors:** Ying E Zhang

**Affiliations:** 1 Laboratory of Cellular and Molecular Biology, Center for Cancer Research, National Cancer Institute, National Institutes of Health, Bethesda, MD, USA

## 

The TGF-β family of cytokines encompasses a large number of versatile poly-peptide growth factors regulating a multitude of cellular processes in almost every aspect of cellular function, including proliferation, differentiation, apoptosis, migration, and adhesion. Inappropriate signaling by these factors is closely linked to cancer and other diseases. Although much has been known about the TGF-β signaling pathway since the discovery of Smads 15 years ago, remarkable progress has been made during the past few years in revealing details of different regulatory mechanisms that afford a full range of control of the biological functions of the TGF-β. In this special review issue of *Cell & Bioscience *[1], Xiaochu Chen and Lan Xu summarize how subcellular localization of Smad proteins is regulated [2], Liu-Ya Tang and Ying Zhang describe non-degradative ubiquitin modification in controlling Smad activity [3], and Xuedong Liu and colleagues review different modes of cross-talk between TGF-β and MAPK pathways during cancer progression [4].

With the deep understanding of the signaling mechanism and the availability of various genetic models, specific physiological and pathological roles of TGF-β/Smad in different cellular context start to emerge. Gangwen Han and Xiao-Jing Wang summarize the differential role of signaling Smads, including Smad2, Smad3 and Smad4, in squamous cell carcinoma [5]. Yan Chen and his colleagues present recent advances in the understanding of the involvement of inhibitor Smad7 in cancer and other types of diseases [6].

This special issue covers a broad, albeit not all-inclusive, aspect of TGF-β signaling and biology. It is my sincere hope that this collection of review articles will bring our readers to the cutting-edge of current development and is helpful in appreciating new breakthroughs in this field.

## Disclaimer

The opinions expressed in this Editorial are of the author's personal view and do not necessarily reflect the views of her employer, the National Institutes of Health, USA.

## References

[B1] Cell & Bioscience http://www.cellandbioscience.com

[B2] Chen X Xu L Mechanism and regulation of nucleocytoplasmic trafficking of Smad Cell & Bioscience 2011 1 40 10.1186/2045-3701-1-40 22204445 PMC3292837

[B3] Tang LY Zhang YE Non-degradative ubiquitination in Smad-dependent TGF-beta signaling Cell & Bioscience 2011 1 41 10.1186/2045-3701-1-41 22204598 PMC3293007

[B4] Chapnick DA Warner L Bernet J Rao T Liu X Partners in Crime: The TGFβ and MAPK pathways in cancer progression Cell & Bioscience 2011 1 42 10.1186/2045-3701-1-42 22204556 PMC3275500

[B5] Han G Wang XJ The role of TGF-β signaling Smads in squamous cell carcinoma Cell & Bioscience 2011 1 43 10.1186/2045-3701-1-43 22204491 PMC3285038

[B6] Zhu L Chen S Chen Y Unraveling the biological functions of Smad7 with mouse models Cell & Bioscience 2011 1 44 10.1186/2045-3701-1-44 22204639 PMC3275527

